# Editorial: From assessment to intervention: role of consumer technology and neurotech in preventive mental health

**DOI:** 10.3389/fpsyg.2024.1372708

**Published:** 2024-02-21

**Authors:** Aamir Saeed Malik, Muhammad Hussain, Safee Ullah Chaudhary

**Affiliations:** ^1^Department of Computer Systems, Faculty of Information Technology, Brno University of Technology, Brno, Czechia; ^2^Department of Computer Science, College of Computer and Information Sciences, King Saud University, Riyadh, Saudi Arabia; ^3^Department of Life Sciences, Syed Babar Ali School of Science and Engineering, Lahore University of Management Sciences, Lahore, Pakistan

**Keywords:** neurotech, consumer technology, preventive, mental health, AI

Modern research has established mental health and wellbeing as a key determinant of an individual's quality of life. Good mental health is also a key determinant of human physical health, and poor mental health can lead to poor physical health as well as aberrant social behavior. Consequently, mental health problems bring with them a plethora of physical conditions as well, such as an increase in obesity levels induced by schizophrenia and depression, thus leading to fatalities from coronary heart disease, etc. People with mental health problems have a median life expectancy loss of 10 years, making these diseases responsible for 14.3% or around 8 million deaths per year worldwide (Walker et al., 2015). In 2019, a World Health Organization (WHO) study showed that 1 in every 8 people in the world was living with mental health problems (Mental Disorders, 2024), as shown in Figure 1. It is a very big number (almost a billion people), and the only way to address it is through preventive mental health. Unfortunately, at the early stage of mental health issues, people themselves are not aware of such issues and hence can't seek help. As the disease progresses and some early signs and symptoms become evident, people avoid talking about it and avoid seeking help for several reasons, including social stigma associated with mental health issues. Therefore, to detect early signs of mental disorders, the mental state needs to be monitored continuously for any symptoms of deterioration using consumer products and neurotech from the comfort of the home, thereby empowering the common person. This Research Topic highlights the importance of using consumer technology for preventive mental health. The articles published in this issue are related to the detection of academic stress and social anxiety disorder, which are some of the core mental health issues that further lead to many disorders like depression and schizophrenia. One of the articles also highlights the importance of online mental health communities.

**Figure 1:**
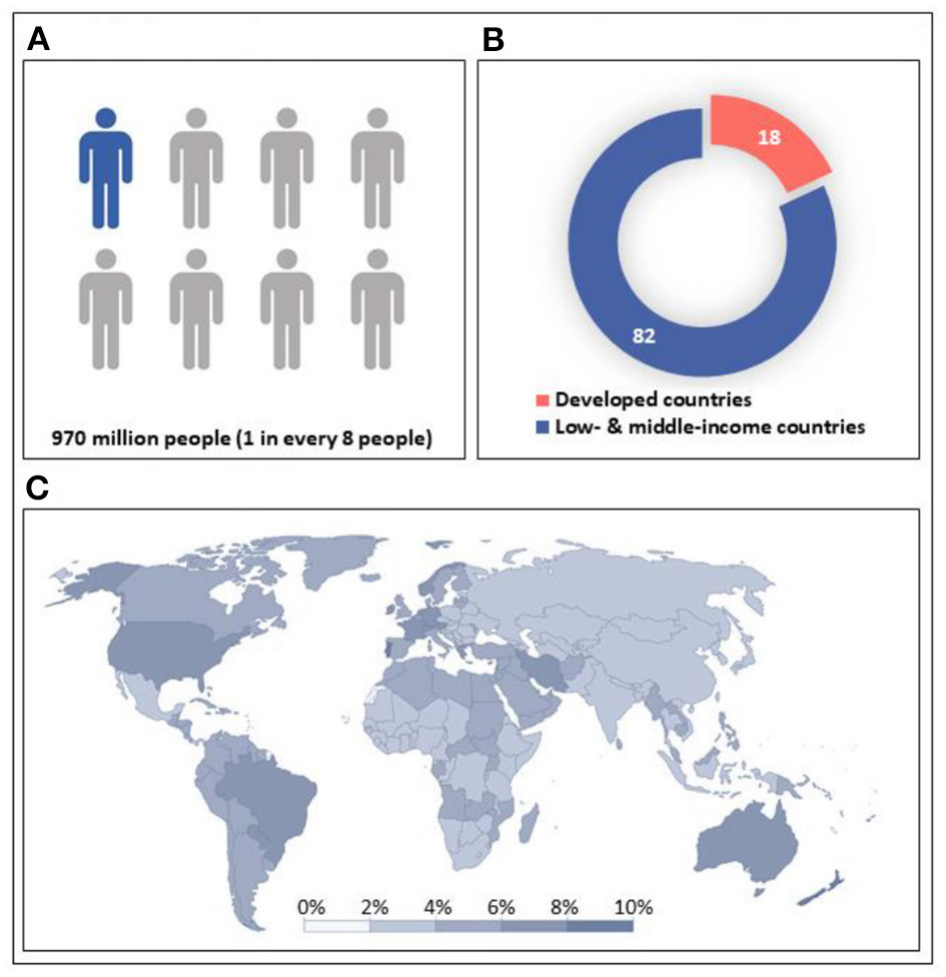
Prevalence of mental health Issues by regions (Walker et al., 2015). **(A)** Global prevalence. **(B)** Prevalence by income group. **(C)** Prevalence by country.

In this Research Topic, Guo et al. expand the understanding of factors influencing the perceived helpfulness of mental health information in online communities by focusing on constructs of information quality and responder's effort. The data was collected from a Chinese online mental health community employing theoretical frameworks of the elaboration likelihood model and motivation theory, with a reputation as an extrinsic motivator. Analysis of 11 key variables, including health information length, readability, response consistency, and user views, employs negative binomial regression. The authors reveal that detailed health information has a positive impact on perceived helpfulness, while easily understandable content may be perceived as less helpful. Timely responses and interactive feedback positively contribute to perceived helpfulness, underscoring their importance in online mental health communities. Al-Ezzi et al. undertake early Social Anxiety Disorder (SAD) diagnosis and severity classification. Employing cerebral information flow patterns and corresponding graphical networks, the proposed methodology categorizes SAD severity into gradations (severe, moderate, mild, and control). Partly directed coherence (PDC) and graph theory measures are used to examine resting-state EEG data. The authors reveal significant PDC disparities between SAD cohorts and healthy controls, particularly in theta and alpha bands. Enhanced information flow is prominent in severe and moderate SAD groups across all bands. This approach advances SAD diagnostic precision, fostering prospects for tailored and effective SAD assessment at home. Lukenga et al. conducted a comprehensive investigation into the perceptions of academic stress management among female university students, with a specific focus on their subjective experiences with technological innovations. The study delves into the diverse array of resources frequently employed by students to alleviate stress, elucidates their contemporary stress reduction needs, and scrutinizes their perspectives on the utilization of digital technologies in the context of academic stress management. The findings of the study reveal a general decline in academic stress over time, emphasizing the advantageous role of social support in ameliorating overall stress levels. Furthermore, the research underscores the superior efficacy of problem-focused coping strategies in addressing academic stress while acknowledging the utility of emotion-focused coping strategies for short-term stress events. The authors conclude by deliberating on the essential attributes of a potential wearable stress management device, advocating for features such as biofeedback and internet connectivity to facilitate tailored and effective coping strategies.

The future of preventive mental health lies in the use of artificial intelligence (AI) for the analysis of data coming from the consumer and neurotech devices. Preventive mental health using consumer technology involves the assessment and detection of early signs and symptoms related to mental health issues. The biomarkers and neuromarkers play a key role in detecting the early signs. The recent developments in machine learning, especially deep learning, have opened opportunities to analyze brain signals and automatically learn the biomarkers and neuromarkers using consumer technology. However, certain challenges must be addressed to adopt machine learning and deep learning. One commonly used approach for learning models is the subject-independent scenario, where the data from the same subject is used for training and validation. It leads to poor generalization and overfitting because, in this scenario, the model also learns the markers related to the identity of subjects rather than learning only the biomarkers indicatives of early signs and symptoms of mental health issues. It is essential that the models must be learned in cross-subject scenarios, where the data from different subjects is used for training and validation. This ensures that the learned biomarkers are associated with mental disorders. However, it is a challenging issue because there is a significant variation in the characteristics of brain signals, such as EEG recorded from different subjects and at different times; that is, there is a covariate shift in characteristics of the data. There is a need to explore the domain adaptation techniques from the learning paradigms in this situation. In addition, there is a problem with the interpretability of the learned biomarkers; to develop confidence about the biomarkers, it is necessary that the learning process that gives rise to the biomarkers must be explainable. Deep learning has shown outstanding performance in detection and diagnosis tasks, but learning deep models in scenarios where the annotated data is limited poses another challenge; one future direction to tackle this issue is to explore few-shot learning.

## Author contributions

AM: Writing—original draft, Writing—review & editing. MH: Writing—original draft, Writing—review & editing. SC: Writing—original draft, Writing—review & editing.
